# Comparison of DNA concentration and bacterial pathogen PCR detection when using two DNA extraction kits for nasopharyngeal/oropharyngeal samples

**DOI:** 10.1371/journal.pone.0289557

**Published:** 2023-08-03

**Authors:** Dam Khan, Shola-Able Thomas, Peggy-Estelle Tientcheu, Sambou M. S. Suso, Christopher Dupont, Brenda Kwambana-Adams, Nuredin Ibrahim Mohammed, Mark P. Nicol, Martin Antonio

**Affiliations:** 1 Medical Research Council Unit The Gambia at the London School of Hygiene & Tropical Medicine, Banjul, The Gambia; 2 J. Craig Venter Institute, La Jolla, CA, United States of America; 3 NIHR Global Health Research Unit on Mucosal Pathogens, Division of Infection and Immunity, University College London, London, United Kingdom; 4 Division of Infection and Immunity, School of Biomedical Sciences, University of Western Australia, Perth, Australia; 5 Centre for Epidemic Preparedness and Response, London School of Hygiene & Tropical Medicine, London, United Kingdom; 6 Department of Infection Biology, Faculty of Infectious and Tropical Diseases, London School of Hygiene & Tropical Medicine, London, United Kingdom; Cairo University Faculty of Veterinary Medicine, EGYPT

## Abstract

**Introduction:**

Several important human pathogens that cause life-threatening infections are asymptomatically carried in the Nasopharynx/Oropharynx (NP/OP). DNA extraction is a prerequisite for most culture-independent techniques used to identify pathogens in the NP/OP. However, components of DNA extraction kits differ thereby giving rise to differences in performance. We compared the DNA concentration and the detection of three pathogens in the NP/OP using the discontinued DNeasy PowerSoil Kit (Kit DP) and the DNeasy PowerLyzer PowerSoil Kit (Kit DPP).

**Methods:**

DNA was extracted from the same set of 103 NP/OP samples using the two kits. DNA concentration was measured using the Qubit 2.0 Fluorometer. Real-time Polymerase Chain reaction (RT-PCR) was done using the QuantStudio 7-flex system to detect three pathogens: *S*. *pneumoniae*, *H*. *influenzae*, and *N*. *meningitidis*. Bland-Altman statistics and plots were used to determine the threshold cycle (Ct) value agreement for the two kits.

**Results:**

The average DNA concentration from kit DPP was higher than Kit DP; 1235.6 ng/ml (SD = 1368.3) vs 884.9 ng/ml (SD = 1095.3), p = 0.002. Using a Ct value cutoff of 40 for positivity, the concordance for the presence of *S*. *pneumoniae* was 82% (84/102); 94%(96/103) for *N*. *meningitidis* and 92%(95/103) for *H*. *influenzae*. Kit DP proportionately resulted in higher Ct values than Kit DPP for all pathogens. The Ct value bias of measurement for *S*. *pneumoniae* was +2.4 (95% CI, 1.9–3.0), +1.4 (95% CI, 0.9–1.9) for *N*. *meningitidis* and +1.4 (95% CI, 0.2–2.5) for *H*. *influenzae*.

**Conclusion:**

The higher DNA concentration obtained using kit DPP could increase the chances of recovering low abundant bacteria. The PCR results were reproducible for more than 90% of the samples for the gram-negative *H*. *influenzae and N*. *meningitidis*. Ct value variations of the kits must be taken into consideration when comparing studies that have used the two kits.

## Introduction

The human nasopharynx and oropharynx (NP/OP) are ecological niches for a diverse number of microbes that play important roles in health and disease [1, 2]. Asymptomatic colonisation of the NP/OP by pathogens is a major risk factor for the development of life-threatening infections [3]. A disruption in the microbial balance in the NP/OP may cause some pathogenic bacteria to shift to other niches such as the lungs where they ultimately cause disease [4]. For example, a comparative analysis of carriage and invasive isolates from pneumonia patients showed similarity in microbial strains recovered from carriage and invasive body sites [5, 6]. Thus, the study of microbial populations in the NP/OP is crucial to disease surveillance and control.

Microbiology culture has long been a method of choice to identify bacteria in the NP/OP in epidemiologic studies. However, recent technological advances have led to the rapid rise in the use of culture-independent techniques such as qPCR and sequencing. Genomic DNA extraction is one of the prerequisite methods of choice to most culture-independent techniques. The extraction kit and procedure employed should ensure the efficient recovery of DNA from diverse bacteria, to reflect the true bacterial composition of the NP/OP. Previous reports suggest that bacterial identification by PCR and sequencing is affected by the choice of extraction procedure [7–10]. Commercially available DNA extraction kits mostly differ in their cell lysis protocols thereby giving rise to differences in performance [9, 11]. Ultimately, these differences could result in challenges in comparative analysis of NP/OP carriage between studies that have utilized different extraction kits.

The QIAGEN DNEASY Powersoil kit (Kit DP) has been widely used for the extraction of DNA from NP/OP for PCR and 16S rRNA amplicon sequencing [12, 13]. However, the kit is now discontinued by the manufacturer. This coincided with the period when a portion of samples of a bacterial carriage study we conducted was processed using the kit. A change of kit was done in the middle of the project as a result. The QIAGEN DNEASY Powerlyzer Powersoil kit (Kit DPP) is an alternative to Kit DP. In this work, we compared the performance of kit DP and Kit DPP in extracting DNA from NP/OP specimens. This was done by comparing DNA concentration and detection of *Streptococcus pneumoniae*, *Neisseria meningitidis* and *Haemophilus influenzae* in DNA samples obtained from Kit DP and Kit DPP.

## Methods

### Sample collection

The NP/OP samples were collected from 103 healthy children aged 5–14 years from the Foni district of The Gambia as part of the Respiratory Microbiota of Gambian Children study (ReMAC). The inclusion criteria were: study participants must not have received antibiotic treatment within 4 weeks of sample collection, and that they do not have existing medical conditions for which they are receiving regular medical treatment at the time of recruitment. Trained nurses obtained the NP/OP samples using flexible nasopharyngeal FLOQSwabs® (Copan Diagnostics, Inc.). The swabs were then placed in 1 ml of skim-milk tryptone glucose glycerol broth (STGG) and stored at -70°C until analysed.

### Ethical approval

All study participants were minors. Therefore, written consent was obtained from their guardians or parents prior to their enrolment in the study. Every guardian was given a copy of the participant information sheet and consent form. The information sheet and consent form were interpreted in vernacular for guardians/parents that were not fluent in English. Furthermore, the study was approved by the Joint Medical Research Council Unit The Gambia at the London School of Hygiene and Tropical Medicine and the Government of The Gambia ethics committee.

### DNA extraction

DNA was extracted in parallel from the same set of 103 NP/OP samples using the two kits. The beads used to facilitate the mechanical disruption of the cell wall differ between the kits. Kit DP uses _∼_ 0.7 mm crushed garnet beads while Kit DPP employs 0.1 mm glass beads. Other reagents including lysis buffer, wash buffer, Sodium Dodecyl Sulfate (SDS)-containing solution, high concentration salt solution and elution buffer are the same for both kits. Furthermore, the extraction protocols used were similar. In brief, 200°l of NP/OP sample stored in Skim-milk tryptone glucose glycerol (STGG) was added to the Power Bead tubes provided (garnet or glass beads for Kit DP and Kit DPP respectively) with 60°l of SDS-containing solution to aid in cell lysis. The solution is provided with the kit and its concentration is unknown. The samples were then vortexed for 15 minutes followed by centrifugation at 10,000 x *g* for 30 seconds. A total of 700°l of the supernatant was then added to a 2 ml tube containing 250°l solution of a patented Inhibitor Removal Technology® (IRT). The mixture was then incubated at 4°C for five minutes. Following centrifugation, 600°l of the supernatant was added to 200°l of a second IRT solution and incubated at 4°C for five minutes to further precipitate inhibitors such as cell debris and proteins. The mixture was then spun down and 750°l of the supernatant was added to 1200°l of a high concentration salt solution provided with the kit to provide the optimal pH conditions for the DNA to bind to the silica column. The resulting solution was loaded to a spin filter and spun to allow the DNA to bind to the silica membrane. A total of 500°l of ethanol based wash buffer was added to the column to remove contaminants while allowing DNA to remain bound to the membrane. The DNA was then eluted in 100°l of elution buffer and then stored at -20°C.

### DNA concentration quantification

DNA concentration was measured using 1°l of extracted DNA on the Qubit 2.0 Fluorometer (Life Technologies, ThermoFisher Scientific, USA) using the Qubit dsDNA HS Assay Kit as per manufacturer’s instruction.

### Real-time polymerase chain reaction

Real-time polymerase chain reaction (rt-PCR) was used for the detection of *S*. *pneumoniae*, *H*. *influenzae*, and *N*. *meningitidis*. Briefly, extracted DNA (2°l) was added to a PCR master mix made up of 12.5°l PerfeCTa MultiPlex qPCR SuperMix (Quantabio), 4.5°l of nuclease-free water, and 2°l of 10°M concentration of each of the forward primer, reverse primer and probe of the three targets. The assays were performed using the Quantstudio 7-flex PCR system. The thermal conditions of the assay were as follow 50°C for 2 min, followed by 95°C for 10 min, and 45 cycles of 95°C for 15 s and 60°C for 1 min. The validation assays for the three targets are described here [14]. The primer/probe sequences used for the three targets are as shown on Table 1.

**Table 1:** Primer/probe sets used for the real-time PCR detection of the three pathogens in the Nasopharynx/Oropharynx.

S/No	*Organism*	Gene target	Primer sequence
1	*Streptococcus pneumoniae*	*lytA*	Forward Primer: 5’-ACGCAATCTAGCAGATGAAGCA-3’
			Reverse primer: 5’-TCGTGCGTTTTAATTCCAGCT-3’
			Probe: 5’-TGCCGAAAACGC”T”TGATACAGGGAG-3’
2	*Neisseria meningitidis*	*SodC*	Forward Primer: 5’GCACACTTAGGTGATTTACCTGCAT-3’
			Reverse primer: 5’-CCACCCGTGTGGATCATAATAGA-3’
			Probe: 5’-CATGATGGCACAGCAACAAATCCTGTTT-3’
3	*Haemophilus influenzae*	*hpd*	Forward Primer: 5’- GGTTAAATATGCCGATGGTGTTG
			Reverse primer: 5’- TGCATCTTTACGCACGGTGTA
			Probe: 5’-TTGTGTACACTCCGT"T"GGTAAAAGAACTTGCAC-3’

### Statistical analysis

Statistical analyses were carried out using Stata 16 software (StataCorp, College Station, Texas). A paired sample t-test was done to determine whether there was a difference between the concentration of DNA extracted from the two kits. A *p*-value of <0.05 was considered significant. Summary statistics and box plots of DNA concentration were also done using Stata 16. Bland Altman plots and statistics were used to measure the degree of cycle threshold (Ct) value agreement of each of *S*. *pneumoniae*, *N*. *meningitidis* and *H*. *influenzae* between the two kits. Ct value is inversely proportional to pathogen copy number. The Blandr package on Rstudio was used to create the Bland Altman plots and perform the Bland Altman statistics.

## Results

### Comparison of DNA concentrations

**Fig 1:**
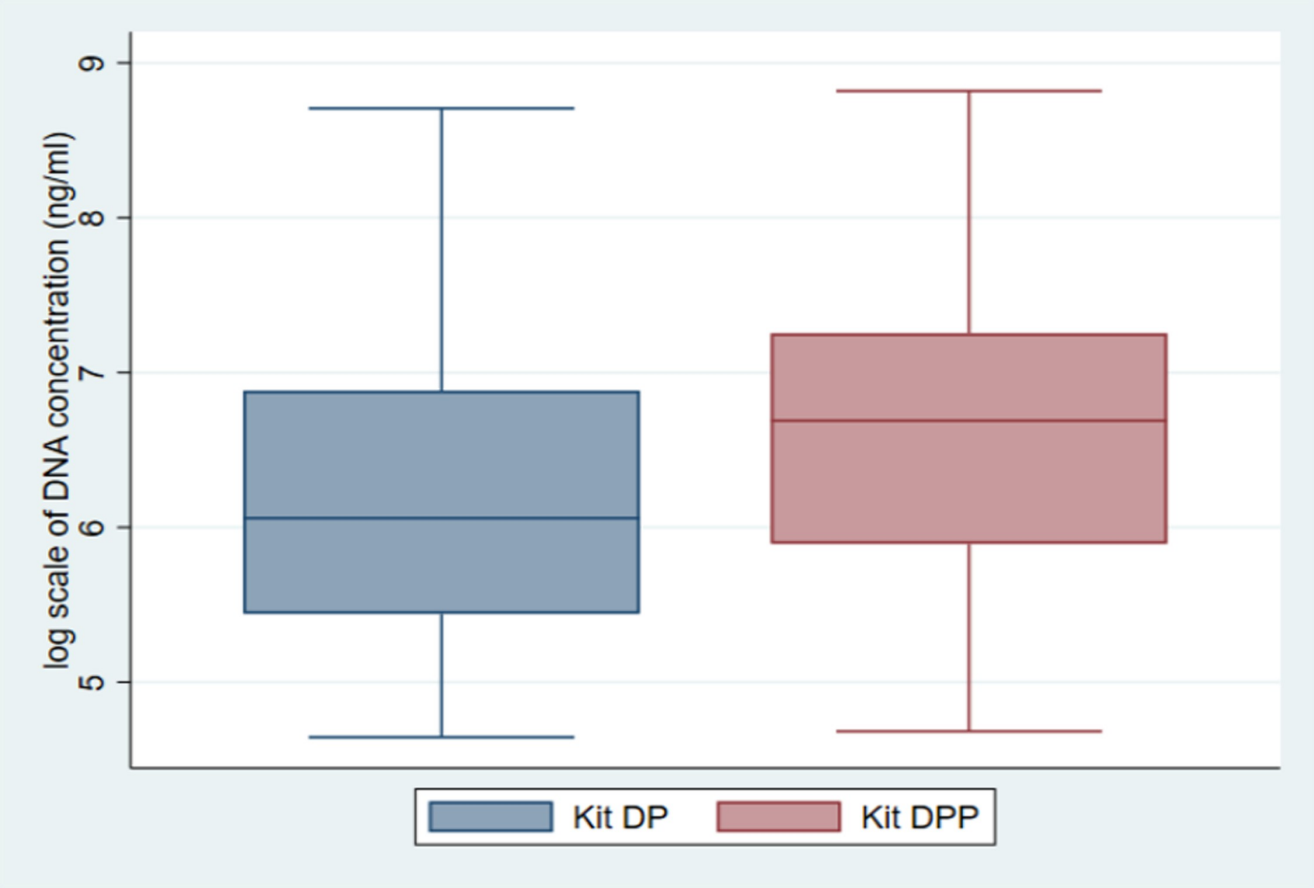
Log scale DNA concentration comparison of kit DP and kit DPP. Samples below the detection limit of the Qubit 2.0 Fluorometer were excluded.

DNA concentrations ranged from 104–6040 ng/ml and 108–6759 ng/ml using kit DP and kit DPP respectively. The paired sample t-test showed a significant difference in DNA concentration between kit DP (mean = 884.9 ng/ml, SD = 1095.3) and Kit DPP (mean = 1235.6 ng/ml, SD = 1368.3) and *p* = 0.002. Seven DNA samples extracted using kit DP and two samples extracted using kit DPP were below the detection limit of the Qubit 2.0 Fluorometer. A box plot of DNA concentration obtained using the two kits is shown in Fig 1.

### Comparison of PCR Ct values obtained using the two kits

The Ct value mean and standard deviation of *S*. *pneumoniae* for Kit DP and kit DPP were 33.2 ± 3.9 and 30.8 ± 3.7 respectively; 35.4 ± 3.6 and 34.0 ± 3.9 for *N*. *meningitidis*, 30.6 ± 3.9 and 29.2 ± 4.0 for *H*. *influenzae*. A Ct threshold of ≤40 has been used in the past to determine positivity in bacterial NP/OP carriage studies [15]. Using a Ct threshold of 40 for positivity, the concordance of the two kits for *S*. *pneumoniae* was 82.4% (84/102); 93.2% (96/103) for *N*. *meningitidis* and 92.2% (95/103) for *H*. *influenzae*. *S*. *pneumoniae* was detected in two samples using kit DP and not kit DPP, and in 16 samples using kit DPP and not Kit DP (Table 2). However, 56.3% (9/16) of *S*. *pneumoniae* positive results recorded using kit DPP and not Kit DP had Ct values ≥36.

**Table 2:** Pathogen detection of the three pathogens using the two extraction kits.

Pathogen	Detection using Kit DP	Detection using Kit DPP	Concordance of the two kits by positivity	Number of positives recorded using Kit DP and not Kit DPP	Number of positives recorded using Kit DPP and not Kit DP
*Streptococcus pneumoniae*	68%(69/102)	81% (83/102)	82% (84/102)	2	16
*Neisseria meningitidis*	41%(42/103)	42% (43/103)	94%(96/103)	2	4
*Haemophilus influenzae*	86%(89/103)	94% (97/103)	92%(95/103)	0	8

Ct value ≤40 used as detection threshold for positivity

**Fig 2:**
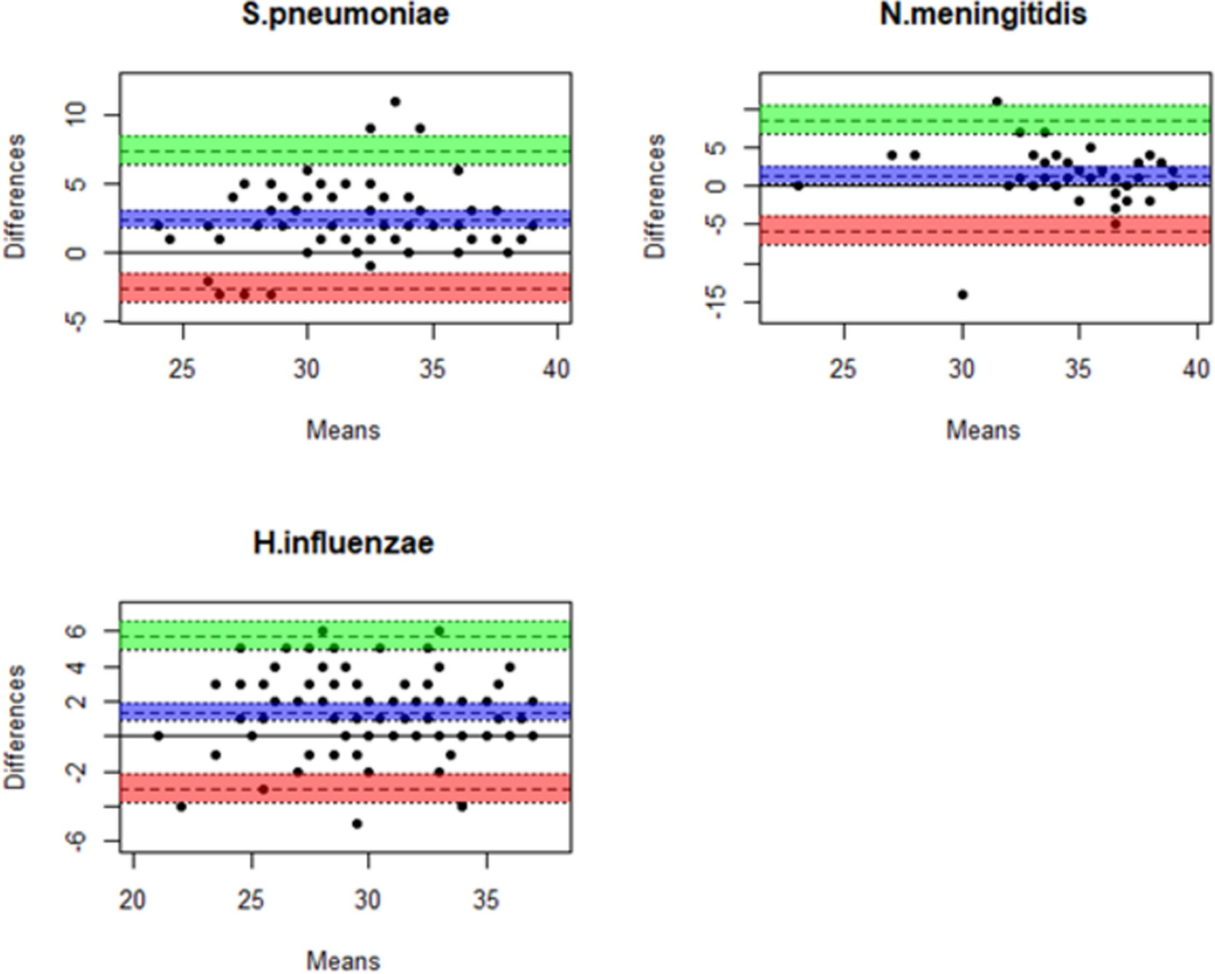
Bland-Altman plots showing Ct value differences and means for paired samples generated using kit DP and kit DPP. The pairwise Ct value differences (Kit DP minus kit DPP) is represented on the Y-axis. The positive data points represent a higher Ct value for Kit DP and negative data points represent a higher Ct value for Kit DPP. The means of paired Ct value measurement is represented on the X-axis. The computed bias is represented by the purple layer and the upper and lower limits of agreement are represented by the green and orange layers respectively.

Bland-Altman plots comparing the PCR Ct values obtained using DNA from the two extraction kits are shown in Fig 2. Positive Bias values indicate a higher average Ct value for kit DP and negative values indicate a higher average Ct value for kit DPP. The bias of measurement for *S*. *pneumoniae* was +2.4 (95% CI, 1.9–3.0); +1.4 (95% CI, 0.9–1.9) for *N*. *meningitidis* and +1.4 (95% CI, 0.2–2.5) for *H*. *influenzae*.

## Discussion

In this article, we compared two QIAGEN DNA extraction kits used for NP/OP samples, with respect to DNA yield and rt-PCR detection of *S*. *pneumoniae*, *N*. *meningitidis and H*. *influenzae*. We showed that Kit DPP yielded higher DNA concentration, more positive PCR results and lower Ct values for *S*. *pneumoniae*, *N*. *meningitidis* and *H*. *influenzae*.

*S*. *pneumoniae* and *H*. *influenzae* are frequent colonizers of the upper airways and are important etiologic agents of bacterial pneumonia [16]. Age, health status and geographic location are among the factors that affect the prevalence of colonization with these pathogens. In Sub-Saharan Africa, *S*.*pneumoniae* nasopharyngeal colonization prevalence could be up to 43% [17–19] among older children and young adults. *N*. *meningitidis* colonization prevalence is the lowest among the three pathogens, but it is the most common etiologic agent of bacterial meningitis in this setting, along with *S*. *pneumoniae* [20]. It should be noted that bacteria in the NP/OP are better identified using qPCR rather than microbiology culture. However, not every bacterium detected by qPCR is viable or transmissible.

Different bead materials with distinct properties are used for cell lysis. Cell lysis efficiency and integrity of molecules extracted are dependent on the size, shape, and molecular composition of the beads used. Small size and spherical-shaped bead materials have been shown to be more effective in extracting parasitic DNA [21]. A similar observation is made in our comparison as KitDPP employs smaller beads than the kit DP.

The dyes used in our qubit assay bind selectively to double-stranded DNA. Since the human genome is vastly larger than bacterial genomes, extracted DNA from human samples is mostly made up of human DNA. Thus, the large percentage of the DNA concentration reading that we obtained was likely contributed by human and not bacterial DNA. Nonetheless, the higher DNA concentration obtained using kit DPP could increase the chances of recovering less abundant bacteria in the NP/OP.

The PCR positivity detection was concordant for more than 90% of the samples for the gram-negative *H*. *influenzae* and *N*. *meningitidis*. The concordance for the gram-positive *S*. *pneumoniae* was lower, with the majority of the disparity observed in samples with a relatively high Ct value for Kit DPP. This could be explained by the fact that Kit DPP is more efficient in extracting DNA of gram-positive bacteria especially when they are represented in low quantities in the sample. The Ct value variations must be taken into consideration when comparing studies that have used the two kits.
